# Supplementary material for: Fine-tuning the pore environment of ultramicroporous three-dimensional covalent organic frameworks for efficient one-step ethylene purification
Source: Nat Commun. 2024 Apr 8;15:3008. doi: 10.1038/s41467-024-47377-3 (PMC11001888; doi:10.1038/s41467-024-47377-3)
Supplement: Supplementary file 1 — Supplementary Information [file 41467_2024_47377_MOESM1_ESM.pdf]

Supplementary Information

Fine-Tuning the Pore Environment of Ultramicroporous Three-Dimensional Covalent Organic Frameworks for Efficient One-Step Ethylene Purification

Yang Xie,[1] Wenjing Wang,[2] Zeyue Zhang,[3] Jian Li,[3,4] Bo Gui,[1] Junliang Sun,[3,*]
Daqiang Yuan,[2,*] and Cheng Wang[1,*]

Supplementary Instruments

^1H and ^{13}C NMR spectra were measured on a Bruker Fourier 400 MHz spectrometer. High-resolution mass spectra were collected on Bruker Solarix. Elemental analysis was conducted on a Flash EA 1112. Fourier transform infrared (FTIR) spectra were recorded on a Nicolet iN10 micro FTIR Spectrometer. Powder X-ray diffraction (PXRD) patterns were obtained on a Rigaku SmartLab X-Ray diffractometer with Cu Kα line focused radiation at 45 kV and 200 mA or Rigaku MiniFlex 600 X-Ray diffractometer. Thermogravimetric analysis (TGA) from 30-800 °C was carried out on a TA-Q500 in nitrogen atmosphere using a 10 °C/min ramp without equilibration delay. Field-emission scanning electron microscope (FE-SEM) was performed on a ZEISS SIGMA operating at an accelerating voltage ranging from 0.1 to 20 kV. The ^{13}C CP MAS spectra were recorded on a Bruker AVANCE NEO 400 WB spectrometer equipped with a 4 mm standard bore. The CP MAS probe head whose X channel was tuned to 100.62 MHz for ^{13}C and the other channel was tuned to 400.18 MHz for broad band ^1H decoupling, using a magnetic field of 9.39 T at 297 K. The dried and finely powdered sample was packed in the ZrO_2 rotor closed with Kel-F cap which was spun at 8 kHz rate. The experiments were conducted at a contact time of 2 ms. A total of 2000 scans were recorded with 3 s recycle delay for each sample. All ^{13}C CP MAS chemical shifts are referenced to the resonances of adamantane standard. UV-vis spectra were recorded on a SHIMADZU UV-3600 UV-vis-NIR spectrophotometer.

The nitrogen sorption isotherms were measured at 77 K by using an Autosorb-iQ2 (Quantachrome) surface area size analyzer. Before measurement, the samples were degassed in a vacuum at 120 °C for 12 h. The Brunauer-Emmett-Teller (BET) surface area was calculated from selected points and pore size distribution was calculated with the NLDFT method.

Supplementary Synthesis

All the raw materials and solvents, unless otherwise noted, were commercially available and used as purchased. Pyridine-2,6-diamine (95%), 4-formylphenylboronic acid (97%), and tetra(*p*-nitrophenyl)methane (97%) were purchased from Adamas.

Synthesis of 2,3,5,6-tetrabromopyridine

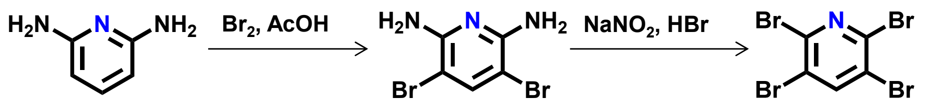

2,3,5,6-tetrabromopyridine was synthesized based on the literature.[1] Bromine (11.4 mL, 220 mmol) was added dropwise into the mixture of pyridine-2,6-diamine (10.9 g, 100 mmol) and acetic acid (200 mL) at 0 °C. Then the reaction system was stirred at room temperature for 5 h. The residual bromine was treated with aqueous Na_2SO_3 solution. And the mixture was neutralized by the addition of aqueous NaOH solution to pH = 9. After filtration, the precipitate was washed with water and dried in vacuo to obtain a brown solid (22 g, 83 %). To a solution of the brown solid (10 g, 37.5 mmol) in 48 % HBr (30 mL) was added dropwise a saturated aqueous solution of $NaNO_2$ (20.7 g, 300 mmol) at 0 °C. The reaction system was then stirred at room temperature for 4 h. The pH of the solution was adjusted to 8 by the addition of aqueous NaOH solution, and the resulting mixture was extracted with ethyl acetate. The organic solution was washed with water, brine, and dried over anhydrous Na_2SO_4. After that, the solvent was evaporated under reduced pressure and the crude product was purified by column chromatography [SiO_2: heptane/dichloromethane = 5/1] to yield the 2,3,5,6-tetrabromopyridine as a white solid (1.3 g, 20 %). ^1H NMR (400 MHz, $CDCl_3$, ppm): δ = 7.99 (s, 1 H).

Synthesis of 2,3,5,6-tetrakis-(4-formylphenyl)pyridine (TPP)

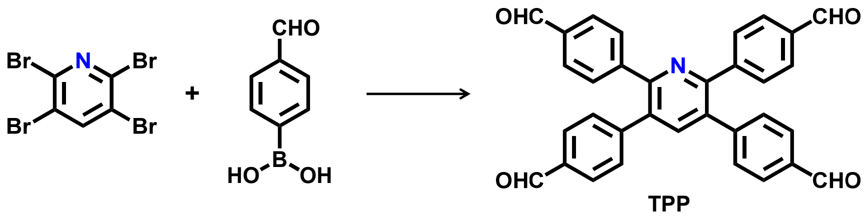

A suspension of 2,3,5,6-tetrabromopyridine (4.00 g, 10.1 mmol), 4-formylphenylboronic acid (12.0 g, 80.0 mmol), $Pd(OAc)_2$ (0.20 g, 0.89 mmol), Sphos (0.80g, 0.19 mmol) and tripotassium orthophosphate (12.0 g, 56.5 mmol) in toluene (150 mL) was heated under nitrogen at 90 °C for 3 days. After cooling to room temperature, the solvent was removed under reduced pressure. Then the residues were dissolved in CH_2Cl_2, washed with water and brine, and dried over anhydrous Na_2SO_4. After that, the solvent was evaporated under reduced pressure and the crude product was purified by column chromatography [SiO_2: CH_2Cl_2/ethylacetate = 40/1] to yield TPP as a white solid (3.70 g, 74% yield). ^{1}H NMR (400 MHz, $CDCl_3$, ppm): δ = 10.04 (s, 2H), 10.02 (s, 2H), 7.90 (s, 1H), 7.87 (d, J = 8.0 Hz, 4H), 7.81 (d, J = 8.0 Hz, 4H), 7.65 (d, J = 8.0 Hz, 4H), 7.46 (d, J = 8.0 Hz, 4H). ^{13}C NMR (100 MHz, $CDCl_3$, ppm): δ = 192.0, 191.7, 155.2, 144.8, 144.7, 144.1, 136.0, 135.7, 134.6, 130.9, 130.3, 130.2, 129.7. HR-MS (MALDI–TOF): calculated for $C_{33}H_{22}NO_4$ m/z = 496.1549 $[M+H]^+$, found: m/z = 496.1548 $[M+H]^+$.

Synthesis of tetra(*p*-aminophenyl) methane (TAPM)

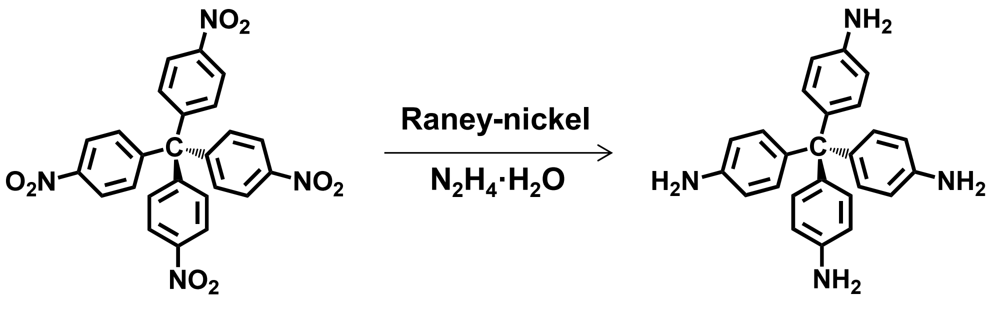

TAPM was synthesized based on the literature.[2] To a mixture of tetra(*p*-nitrophenyl)methane (1.5 g, 2.99 mmol), Raney-nickel (10 g) and THF (100 mL), hydrazine monohydrate (10.0 g) were added dropwise and refluxed for 3 hours. After cooling to room temperature, the mixture was filtered and solvent was evaporated under reduced pressure. The crude product was washed with ethanol and dried to obtain TAPM as a white solid (0.7 g, 61% yield). ^{1}H NMR (400 MHz, DMSO-d_6, ppm): δ = 6.65 (d, J = 8.7 Hz, 8H), 6.35 (d, J = 8.7 Hz, 8H), 4.80 (s, 8H).

Supplementary Discussion

Section 1. Characterization of 3D-TPP-COF

A. FT-IR Spectroscopy Analysis

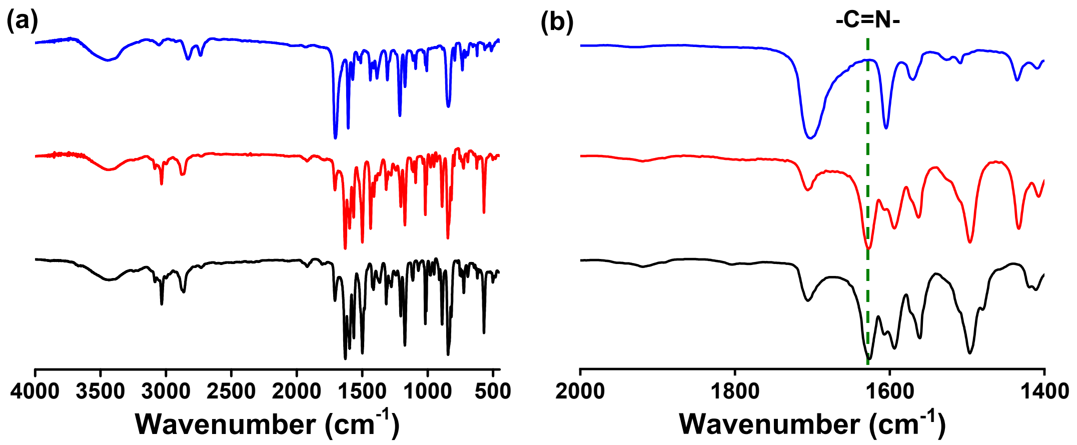

Supplementary Figure 1 Full (a) and enlarged (b) FT-IR spectra of 3D-TPB-COF-H (black curve), 3D-TPP-COF (red curve) and TPP (blue curve).

B. ^{13}C Solid-State NMR Spectroscopy

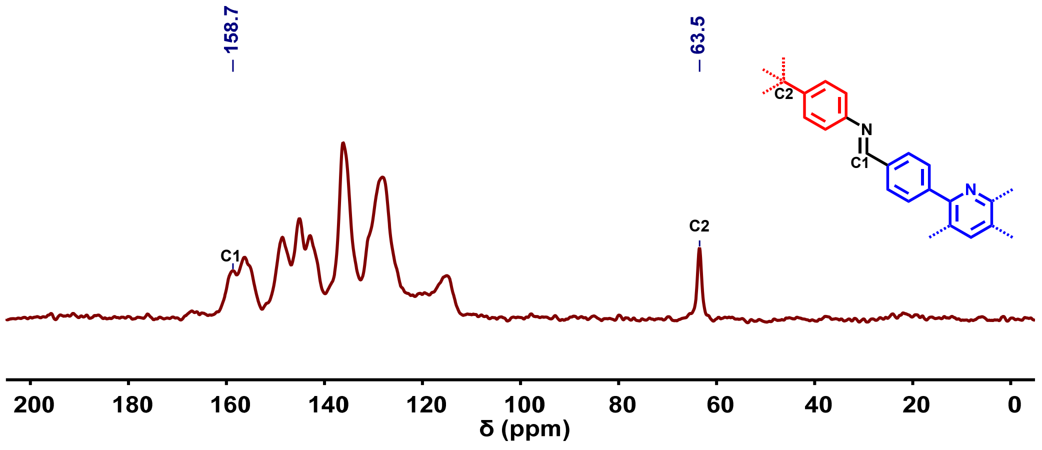

Supplementary Figure 2 ^{13}C solid-state NMR spectrum of 3D-TPP-COF.

C. Scanning electron microscopy

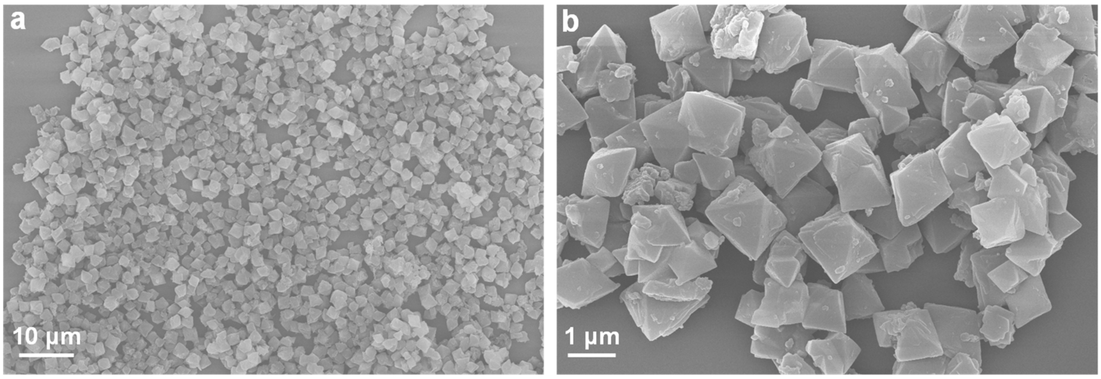

Supplementary Figure 3 SEM images of the 3D-TPP-COF with different scales (scale bar: 10 μm for a and 1 μm for b).

D. UV-vis Absorption Spectrum Study

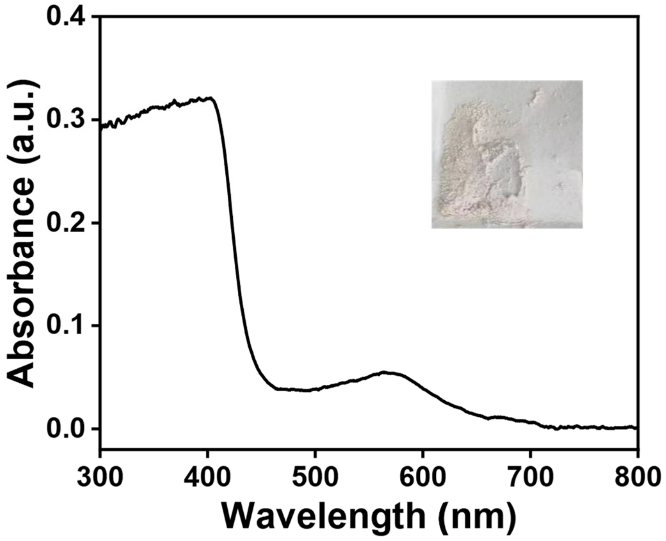

Supplementary Figure 4 Solid-state UV-vis spectrum of 3D-TPP-COF (inset: the picture of 3D-TPP-COF)

E. Thermogravimetric Analysis

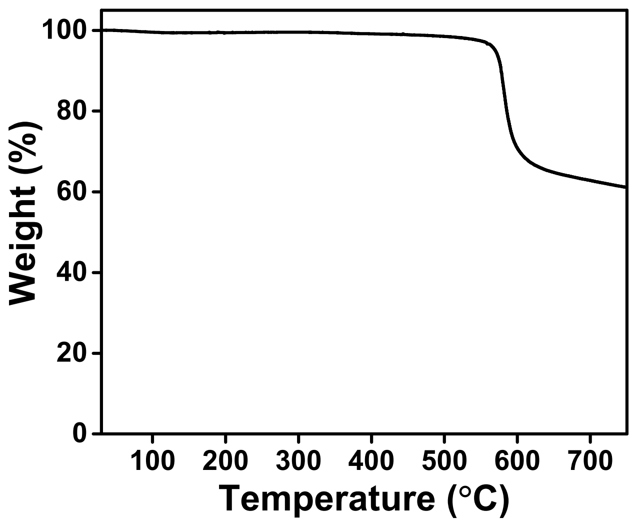

Supplementary Figure 5 TGA profile of the 3D-TPP-COF in nitrogen atmosphere.

F. Chemical Stability

In a general process, 10 mg of 3D-TPP-COF was immersed into different solvents (DMF, DMSO, 1,4-dioxane, ethanol, toluene, acetonitrile, 10^{-2} M HCl and 12 M NaOH) for 24 h at room temperature. Then the powder was filtrated, washed with tetrahydrofuran and dichloromethane and dried under vacuum to measure PXRD patterns. Data were collected on Rigaku MiniFlex 600 X-Ray diffractometer.

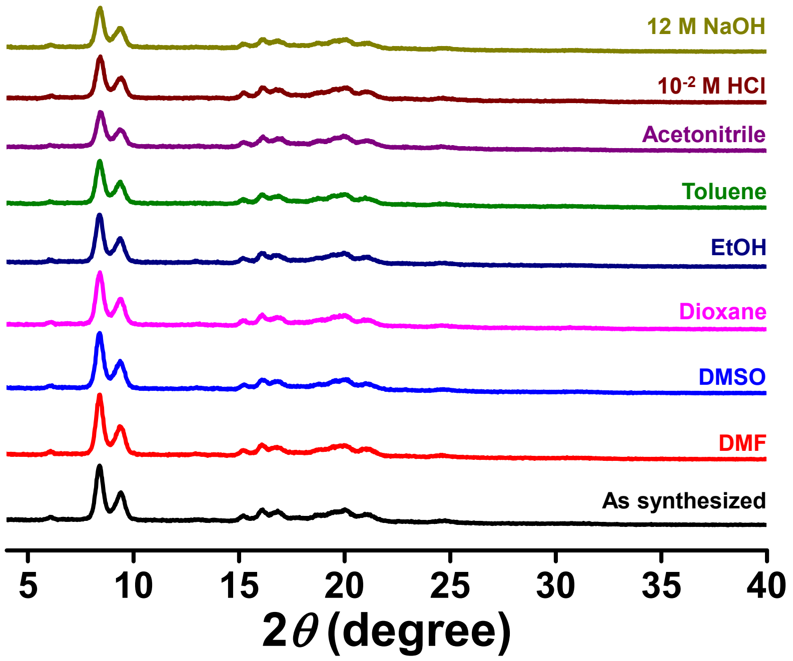

Supplementary Figure 6 PXRD patterns of 3D-TPP-COF before and after being treated in different solvents for 24 h.

G. N_2 sorption experiments

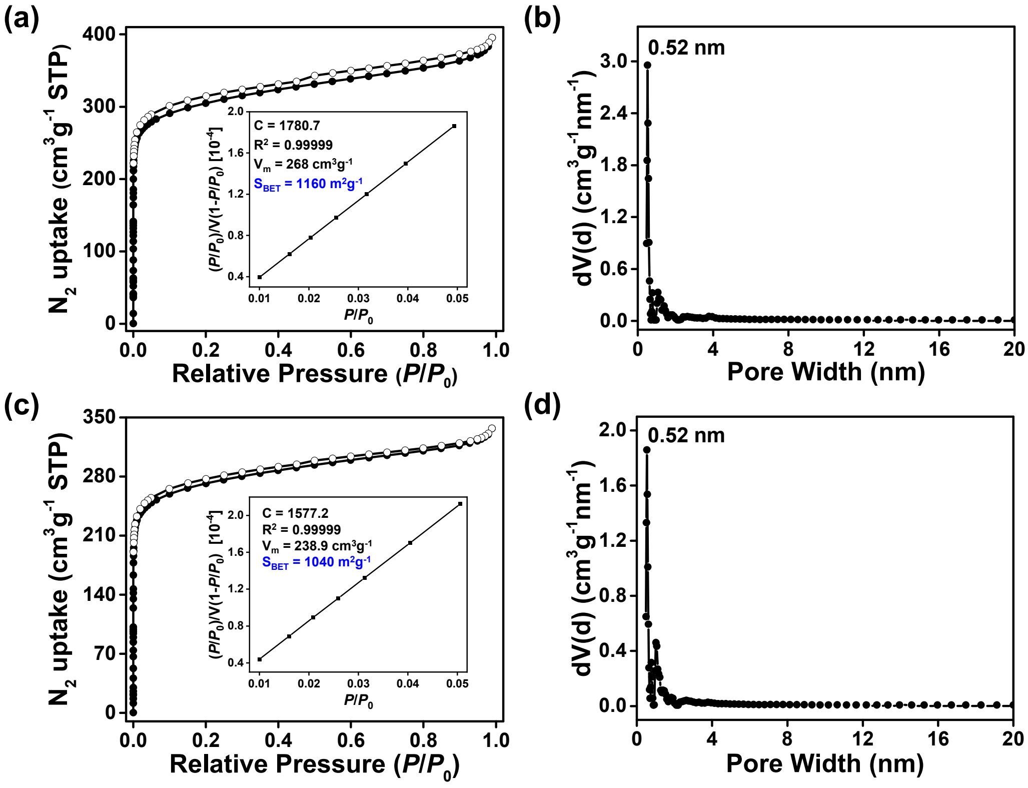

Supplementary Figure 7 The corresponding nitrogen sorption isotherms (inset: selected linear plots) and pore size distributions of 3D-TPP-COF (a, b) and 3D-TPB-COF-H (c, d).

Section 2. Crystal structure analysis

The method of cRED data collection was similar with our previous report.[3,4] Shortly, the sample was cooled down to 99 K by using Gatan cryo-transfer tomography holder and the data was collected by a quad hybrid pixel detector (Timepix) with video mode, using the software of *instamatic*.[5] All the ED patterns were recorded under the spot size 3 with the exposure time 0.5 s. Data processing was conducted using the software package XDS[6] and REDp.[7] Structure solutions were performed by SHELXT[8] with the merged and scaled datasets. PXRD patterns were obtained on a Rigaku SmartLab X-Ray diffractometer with Cu Kα line (λ = 1.5418 Å) focused radiation at 45 kV and 200 mA. The Rietveld refinement with rigid-body constraints was performed on the experimental PXRD using Topas 5.[9]

Supplementary Table 1 Fractional atomic coordinates and the unit cell of 3D-TPP-COF from the Rietveld refinement.

3D-TPP-COF	Space Group: *C*2/*c*<br>a = 28.691(9) Å, b = 7.415(6) Å, c = 27.857(5) Å,<br>$\alpha = \gamma$ = 90°, β = 95.651(4)°,			
Atom	S.O.F.	x/*a*	y/*b*	z/*c*
C1	1	0.03296	0.05062	0.2531
C2	1	-0.02784	-0.20491	0.28145
C3	1	-0.07736	-0.20436	0.28023
C4	1	-0.07574	-0.4616	0.33326
C5	1	0.06248	0.16867	0.25948
C6	1	-0.00315	-0.34137	0.30827
C7	1	-0.10064	-0.32940	0.30630
N8	1	0.11703	0.43846	0.36323
C9	1	0.03216	0.0673	0.33211

C10	1	0.09021	0.30621	0.33517
C11	1	0.0612	0.19071	0.35815
N12	1	-0.10212	-0.59114	0.35734
C13	1	-0.02658	-0.46681	0.3342
C14	1	0.09129	0.29204	0.28558
C15	1	0.22473	1.12072	0.47241
C16	0.5	0.2569	1.22806	0.45393
N16	0.5	0.2569	1.22806	0.45393
C17	1	0.21579	1.14522	0.52046
C18	1	0.20124	0.98121	0.4409
C19	1	0.18333	1.04042	0.54439
C20	1	-0.19898	-0.9418	0.41029
C21	1	-0.16576	-0.83911	0.38938
C22	1	-0.11938	-0.82679	0.41106
C23	1	-0.10655	-0.92277	0.45329
C24	1	0.1367	1.03366	0.52343
C25	1	0.17565	1.03302	0.39814
C26	1	0.15366	0.90388	0.36764
C27	1	0.157	0.72065	0.3793
C28	1	0.18355	0.6678	0.42144
C29	1	0.20582	0.79689	0.45172
C30	1	-0.08631	-0.70635	0.38968
C31	1	0.13205	0.58922	0.347
H32	1	0.37435	0.38442	0.51041

H33	1	0.32407	0.73642	0.35426
H34	1	0.26346	0.55192	0.3927
H35	1	0.43084	0.58613	0.47125
H36	1	0.43685	0.33549	0.78136
H37	1	0.43846	0.30144	0.60095
H38	1	0.49241	0.52057	0.64817
H39	1	0.38418	0.11839	0.7337
H40	1	0.14041	0.32312	0.69452
H41	1	0.0988	0.09999	0.74244
H42	1	-0.03657	0.35013	0.69105
H43	1	0.00575	0.57433	0.6437
H44	1	0.3674	0.55311	0.66732
H45	1	0.32733	0.3182	0.61199
H46	1	0.27196	0.74864	0.51426
H47	1	0.31297	0.98131	0.56891
H48	0.5	0.26295	0.21699	0.4142
C49	1	0	-0.07774	0.25

Section 3. Gas adsorption and separation experiments

Gas adsorption measurements. Automatic volumetric adsorption equipment (Micromeritics, ASAP 2020) was utilized to conduct gas sorption measurements of 3D-TPP-COF and 3D-TPB-COF-H. Before measurements, samples were degassed at 120 °C for 10 h under vacuum. The isosteric heat of sorption for C2 hydrocarbons was regarded as a function of gas uptake using compared the adsorption isotherms at 273, 283 and 293 K. After data being modeled with a virial-type expression comprising a_i and b_i parameters (Eq. (1)), the heat of adsorption (Q_{st}) is determined through fitting parameters by means of Eq. (2), in which *P* refers to the pressure, *N* refers to the adsorbed amount, *T* refers to the temperature, *R* refers to universal gas constant, while *m* and *n* determine the number of terms required to describe the isotherm adequately. The parameters were obtained from fitting of C2 hydrocarbons adsorption isotherms fitted with $R^2 > 0.999$. To assess C_2H_6/C_2H_4 separation performance, IAST of Myers and Prausnitz and pure component isotherm fits by single-site Langmuir–Freundlich equation were employed for calculating molar loadings in the mixture for specific partial pressures of bulk gas phase (Eq. (3)), where *N* refers to molar loading of species (mmol g^{-1}), *A* refers to saturation capacity of species (mmol g^{-1}), *B* refers to Langmuir constant (kPa^{-c}), *C* refers to Freundlich constant and *P* refers to bulk gas phase pressure of species (kPa). The adsorption selectivity based on IAST for C_2H_6/C_2H_4 mixture is identified using Eq. (4):

$$\ln p = \ln N + \frac{1}{T}\sum_{i=0}^{m} a_i N^i + \sum_{i=0}^{n} b_i N^i \qquad (1)$$

$$Q_{st} = -R\sum_{i=0}^{m} a_i N^i \qquad (2)$$

$$N = A\frac{B\times P^C}{1+B\times P^C} \qquad (3)$$

$$S_{A/B} = \frac{x_A y_B}{x_B y_A} \qquad (4)$$

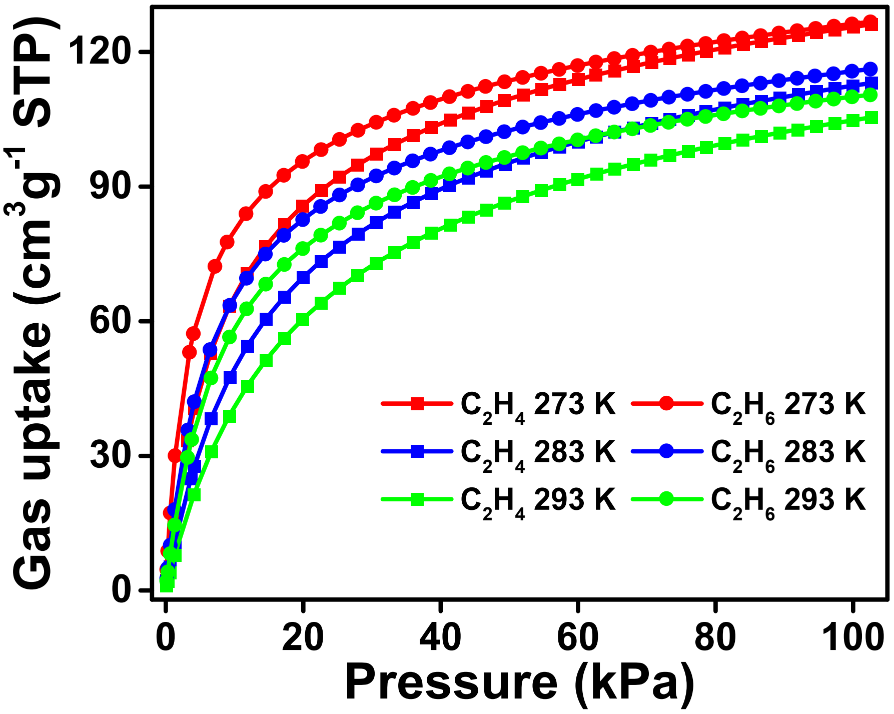

Supplementary Figure 8 Single-component adsorption isotherms of 3D-TPP-COF at 273, 283 and 293 K.

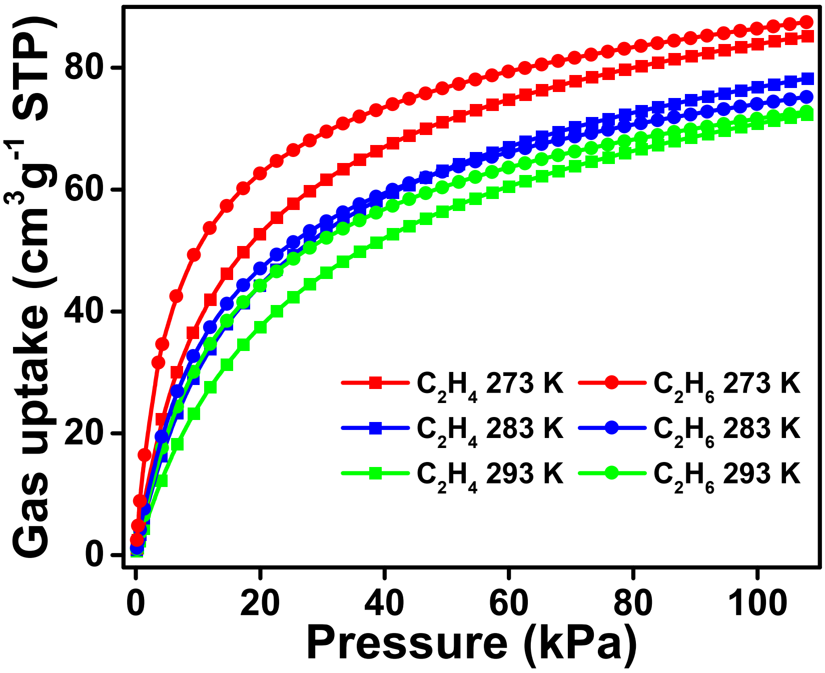

Supplementary Figure 9 Single-component adsorption isotherms of 3D-TPB-COF-H at 273, 283 and 293 K.

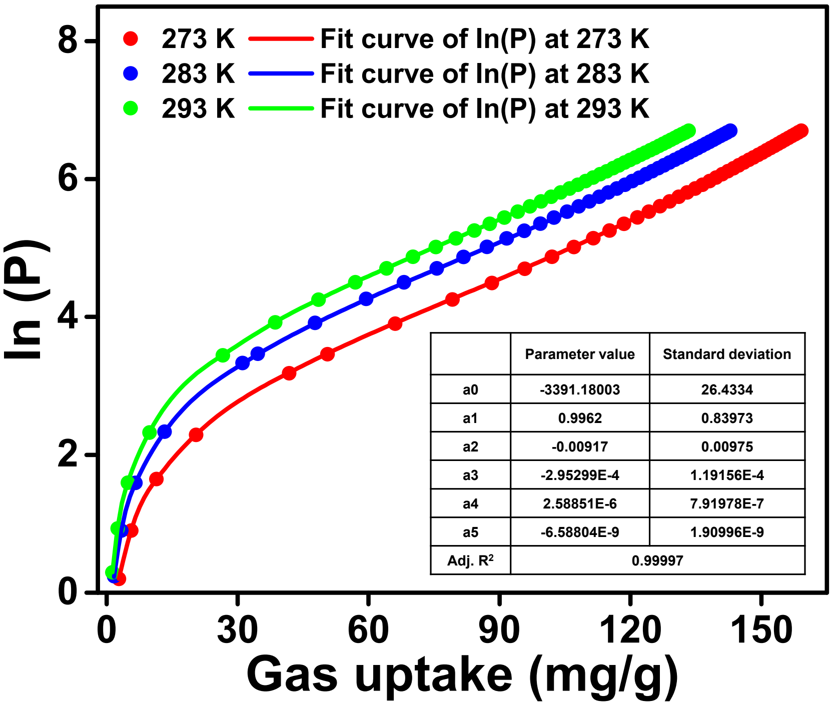

	Parameter value	Standard deviation
a0	-3391.18003	26.4334
a1	0.9962	0.83973
a2	-0.00917	0.00975
a3	-2.95299E-4	1.19156E-4
a4	2.58851E-6	7.91978E-7
a5	-6.58804E-9	1.90996E-9
Adj. R^2	0.99997	

Supplementary Figure 10 Virial equation fitting of the C_2H_4 adsorption isotherm of 3D-TPP-COF at 273, 283 and 293 K.

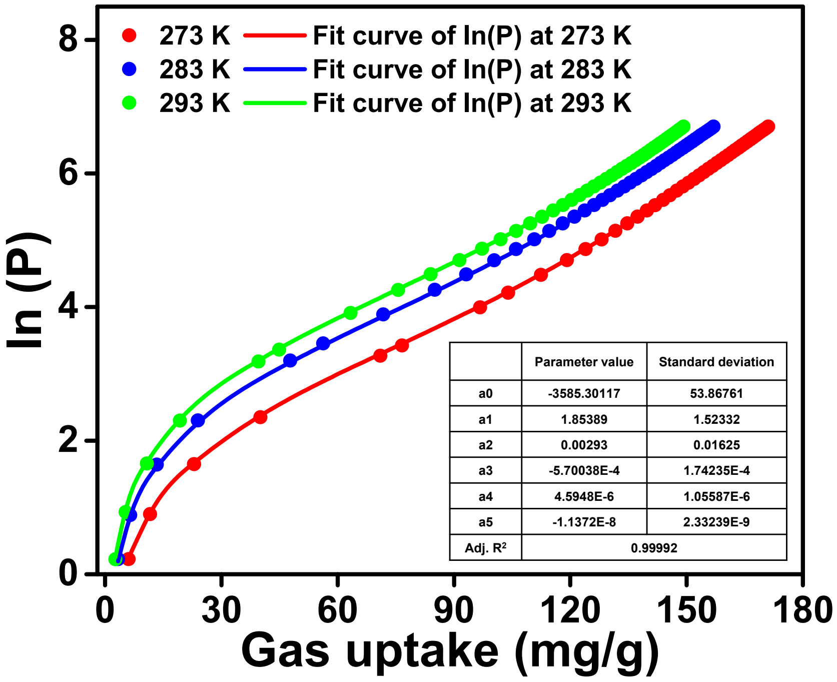

	Parameter value	Standard deviation
a0	-3585.30117	53.86761
a1	1.85389	1.52332
a2	0.00293	0.01625
a3	-5.70038E-4	1.74235E-4
a4	4.5948E-6	1.05587E-6
a5	-1.1372E-8	2.33239E-9
Adj. R^2	0.99992	

Supplementary Figure 11 Virial equation fitting of the C_2H_6 adsorption isotherm of 3D-TPP-COF at 273, 283 and 293 K.

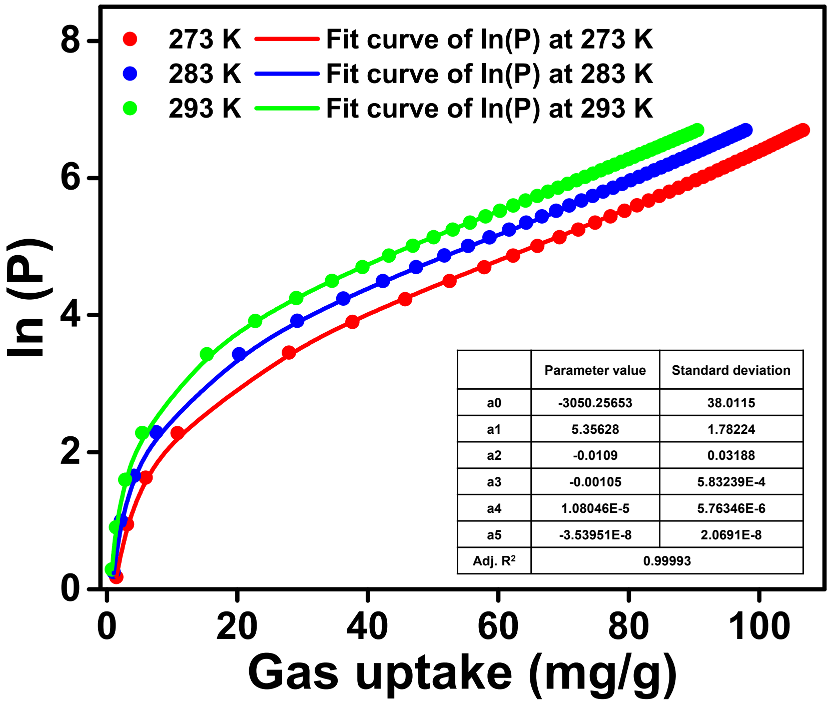

Supplementary Figure 12 Virial equation fitting of the C_2H_4 adsorption isotherm of 3D-TPB-COF-H at 273, 283 and 293 K.

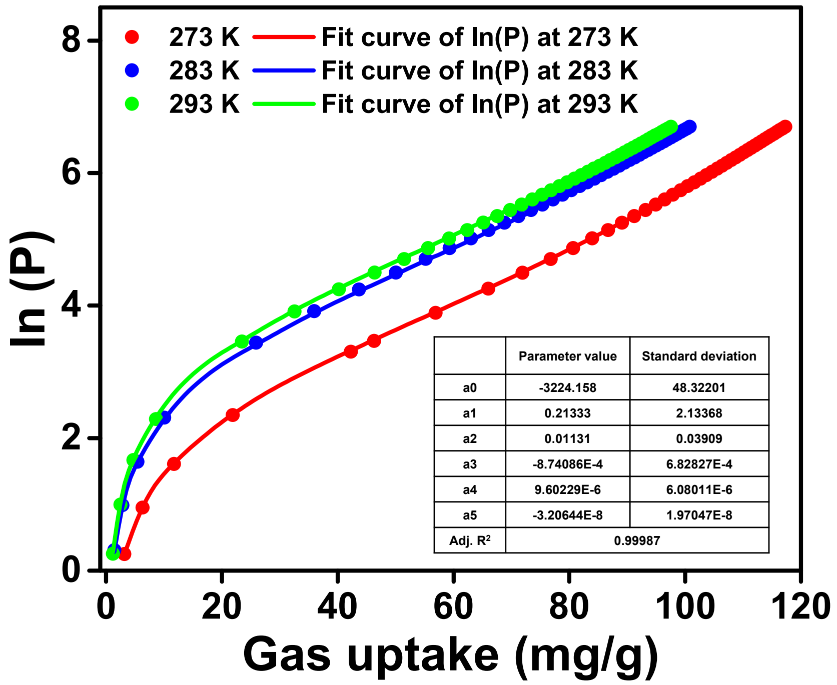

Supplementary Figure 13 Virial equation fitting of the C_2H_6 adsorption isotherm of 3D-TPB-COF-H at 273, 283 and 293 K.

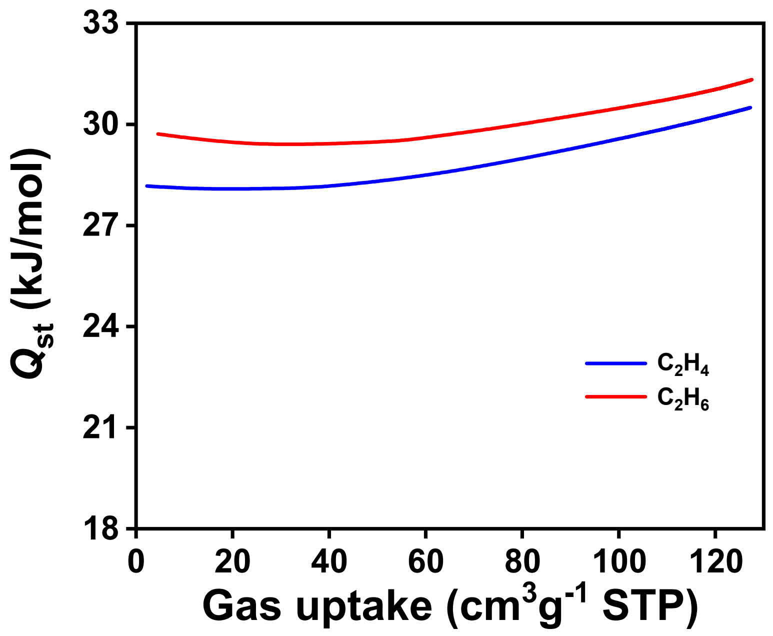

Supplementary Figure 14 The adsorption enthalpies (Q_{st}) of C_2H_6 (red) and C_2H_4 (blue) for 3D-TPP-COF.

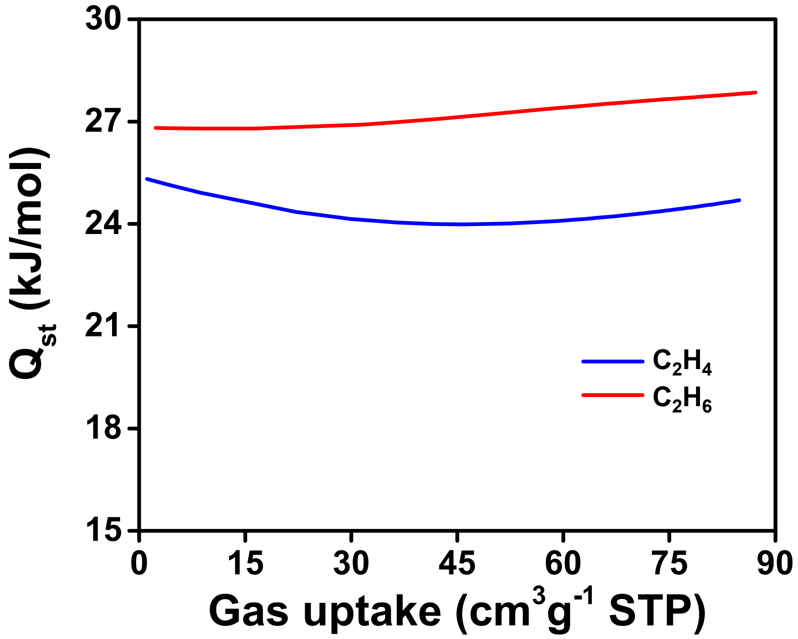

Supplementary Figure 15 The adsorption enthalpies (Q_{st}) of C_2H_6 (red) and C_2H_4 (blue) for 3D-TPB-COF-H.

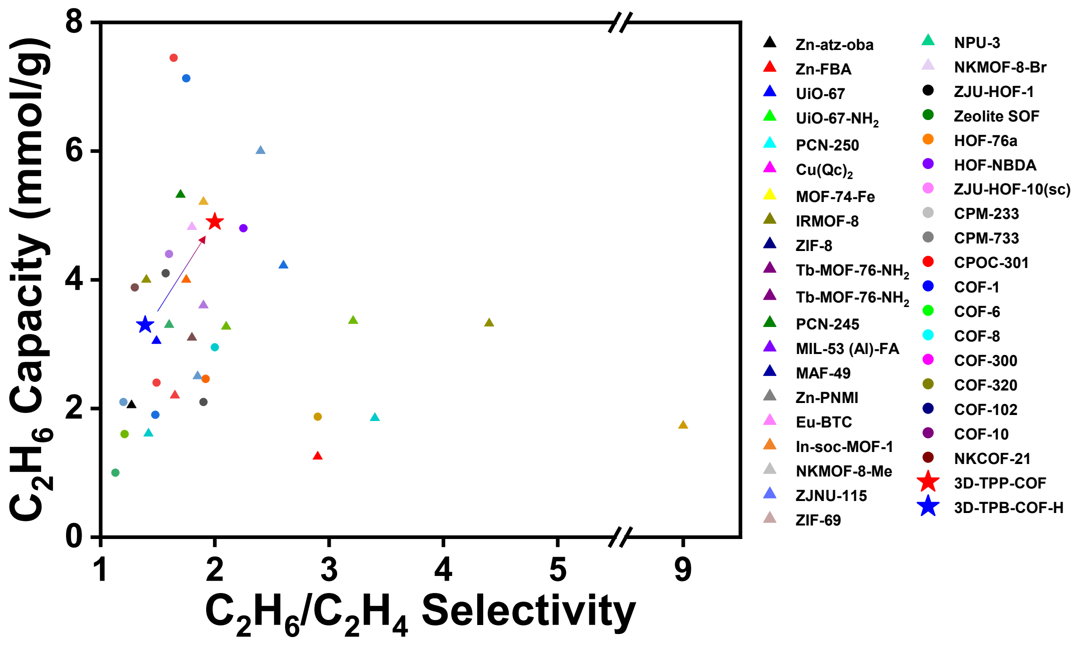

Supplementary Figure 16 Comparison of C_2H_6 capacity and C_2H_6/C_2H_4 selectivity for porous C_2H_6-selective adsorbents at ambient condition.[10] Selectivity values refer to IAST selectivity for a 50/50 C_2H_6/C_2H_4 mixture.

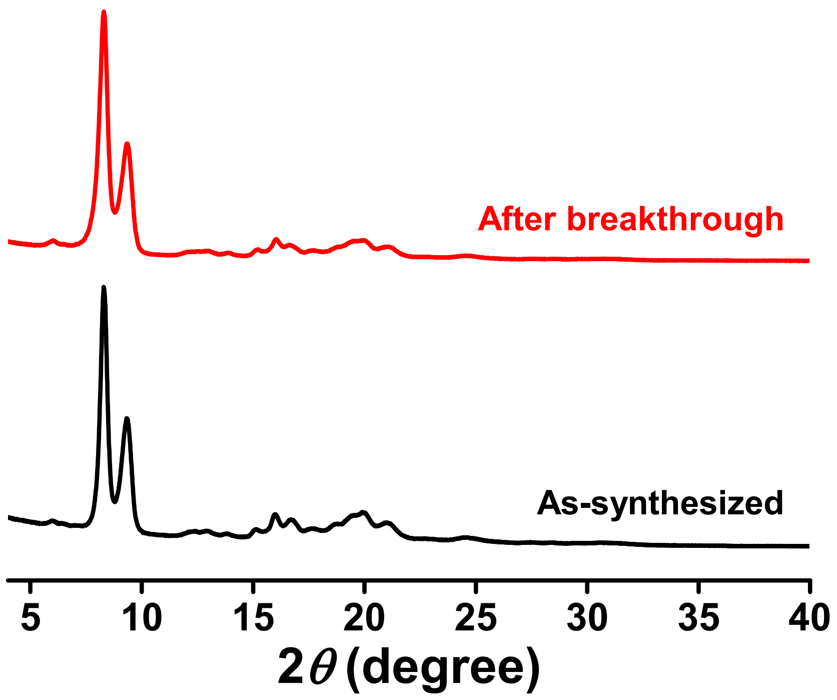

Supplementary Figure 17 PXRD of the 3D-TPP-COF after breakthrough experiments.

Supplementary Table 2 Summary of reported materials for C_2H_6/C_2H_4 separation.

**Materials**	**T (K)**	**C_2H_4**		**C_2H_6**		**IAST Selectivity (C_2H_6/C_2H_4, 1/1)**	**Ref.**
		**Uptake (mmol/g)**	**Q_{st} (kJ/mol)**	**Uptake (mmol/g)**	**Q_{st} (kJ/mol)**		
Zn-FBA	298	1.14	39.8	1.25	42.8	2.9	11
UiO-67-NH_2	298	4.32	24.5	5.32	26.5	1.7	12
Cu(Qc)$_2$	298	0.78	25.4	1.85	29.0	3.4	13
$Fe_2(O_2)$(dobdc)	298	2.60	36.5	3.32	66.8	4.4	14
PCN-250	298	4.22	21.1	5.21	23.2	1.9	15
MAF-49	298	1.70	48.0	2.70	60.0	1.7	16
MUF-15	293	4.15	28.2	1.96	29.2	4.7	17
COF-1	298	1.92	21.5	2.46	22.5	1.9	18
NKCOF-21	298	3.32	23.6	4.37	26.2	1.6	19
NKCOF-22	298	1.82	24.1	2.94	25.9	1.5	
NKCOF-23	298	2.28	23.0	2.70	24.3	1.3	
**3D-TPB-COF-H**	**293**	**3.23**	**25.3**	**3.25**	**26.8**	**1.4**	**This work**
**3D-TPP-COF**	**293**	**4.70**	**28.1**	**4.93**	**29.7**	**1.8**	**This work**

Section 4. Binding energy calculations

From the crystal structure, due to the different orientations of pyridine, we generated two models of 3D-TPP-COF (Figure S16). The initial location of the C2 hydrocarbon guest molecule (one guest molecule per cell) in the unit cell of 3D-TPP-COF was obtained from the classical simulated annealing technique using classical force field as implemented in sorption module in Materials Studio.[20] The free 3D-TPP-COF, C2 hydrocarbon guests, and C2@3D-TPP-COF were further optimized by DFT-D3 calculations performed in CP2K[21] with the PBE functional,[22] DZVP-MOLOPT-SR-GTH basis sets,[23] GTH-PBE pseudopotential,[24] a plane wave grid cutoff of 400 Ry, and the Grimme-D3 dispersion correction.[25] The optimizations converged when the energy difference was less than 1.0×10^{-6} a.u. The inner SCF convergence for each optimization step followed the same criteria along with CP2K defaults for root mean square and max displacement criteria. The binding energies (ΔE bind in kJ mol−1) were calculated as the differences in total energies E between fully optimized C2@3D-TPP-COF and the 3D-TPP-COF and C2 hydrocarbon guests in terms of the following equation:

$$\Delta \boldsymbol{E} = \boldsymbol{E}_{\boldsymbol{COF+gas}} - \boldsymbol{E}_{\boldsymbol{COF}} - \boldsymbol{E}_{\boldsymbol{gas}}$$

where $E_{COF+gas}$ stands for the energy of the fully optimized C2@3D-TPP-COF structure, while E_{COF} and E_{gas} respectively represent energies of bare 3D-TPP-COF structure and isolated C2 hydrocarbon molecule, respectively. Based on such an equation, more negative binding energy means more favorable binding.

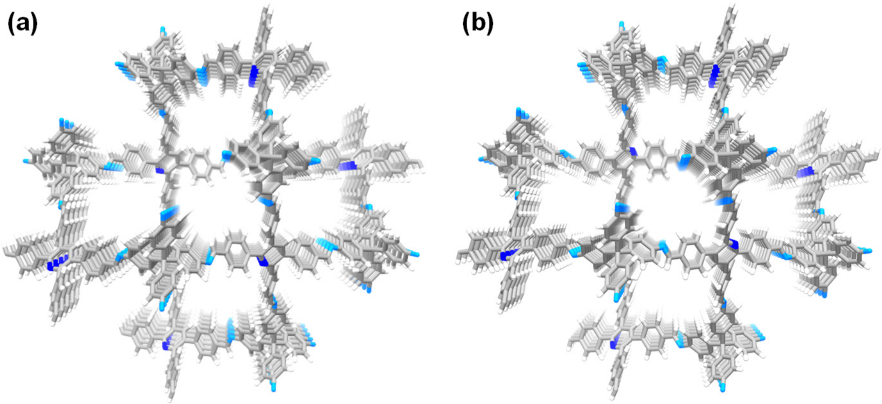

Supplementary Figure 18 Two structure models of 3D-TPP-COF with different pore environments due to the different orientations of pyridine: configuration-A (a) and configuration-B (b). For clarity, the pyridinyl nitrogen is shown in blue, and nitrogen in imine is shown in sky blue.

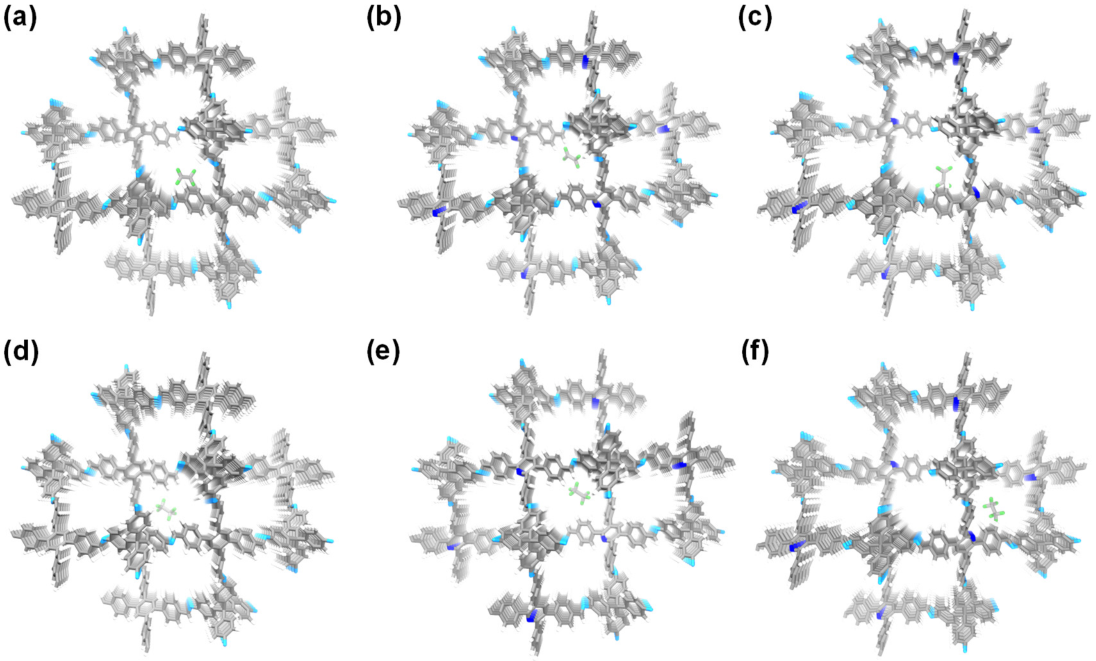

Supplementary Figure 19 Calculated first adsorption sites of C2 gases in 3D COFs. C_2H_4 in 3D-TPB-COF-H (a) and 3D-TPP-COF [configuration-A (b) and configuration-B (c)]; C_2H_6 in 3D-TPB-COF-H (d) and 3D-TPP-COF [configuration-A (e) and configuration-B (f)].

Supplementary Table 3 The binding energies (ΔE) for C2@3D COFs calculated by the Dmol3 module.

	**3D-TPB-COF-H**	**3D-TPP-COF**	
		**configuration-A**	**configuration-B**
**E_{COF} (ha)**	-1591.9790	-1606.5995	-1606.5829
**E_{C2H4} (ha)**	-13.7226		
**$E_{C2H4@COF}$ (ha)**	-1605.7177	-1620.3392	-1620.3240
**$\Delta E_{C2H4@COF}$ (ha)**	-0.0161	-0.0171	-0.0185
**$\Delta E_{C2H4@COF}$ (kJ/mol)**	-42.22	-44.79	-48.65
**E_{C2H6} (ha)**	-14.9543		
**$E_{C2H6@COF}$ (ha)**	-1606.9503	-1621.5706	-1621.5582
**$\Delta E_{C2H6@COF}$ (ha)**	-0.0170	-0.0168	-0.0210
**$\Delta E_{C2H6@COF}$ (kJ/mol)**	-44.70	-44.19	-55.46

Supplementary Table 4 The interactions between the gas molecules and host framework in 3D-TPB-COF-H and 3D-TPP-COF-B.

		3D-TPB-COF-H		3D-TPP-COF	
		H···A (Å)		H···A (Å)	
C_2H_4	C–H···N	2.80	3.37	2.55	3.84
		3.76	3.98	2.68	4.00
				4.23	
	C–H···π	3.64	3.71	3.22	3.71
		4.14	4.17	4.13	4.19
		4.34	4.47	4.33	
C_2H_6	C–H···N	2.88	3.74	2.90	3.81
		3.88	3.98	3.83	4.15
		4.09		4.18	4.45
				4.46	
	C–H···π	3.34	3.65	3.69	3.97
		4.09	4.17	3.74	4.01
		4.23	4.45		

Section 5. NMR Spectra

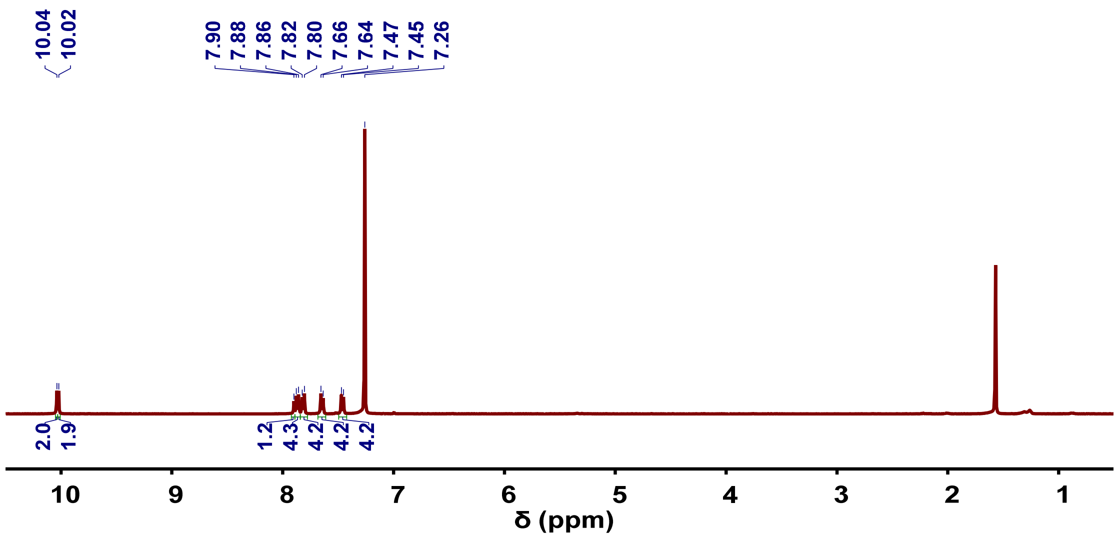

Supplementary Figure 20 ^{1}H NMR spectrum ($CDCl_3$, 400 MHz) of TPP.

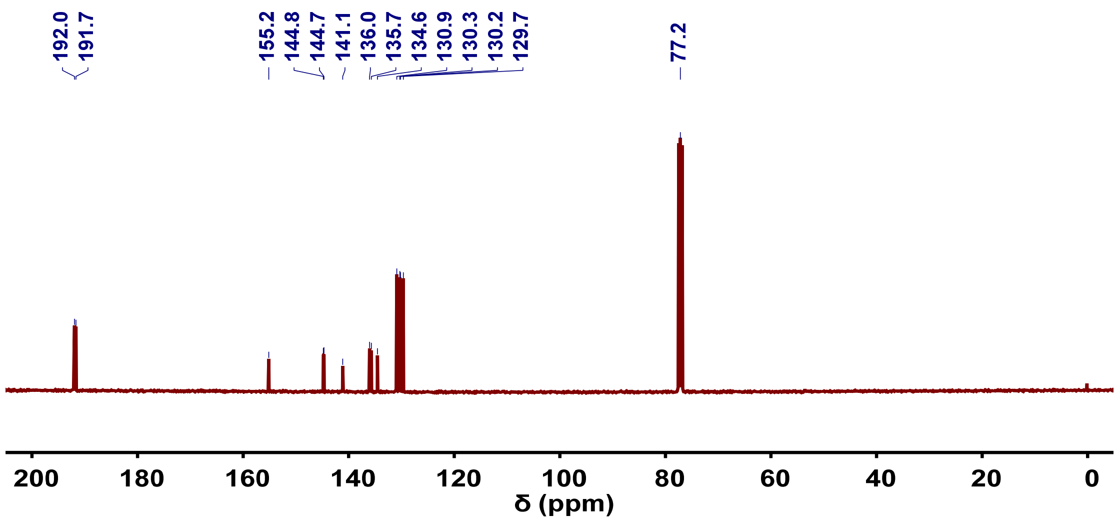

Supplementary Figure 21 ^{13}C NMR spectrum ($CDCl_3$, 100 MHz) of TPP.

Supplementary References

1. Chen, T. K. & Flowers, W. T. A convenient synthesis of 2,3,5,6-tetrahalogenopyridines and of 3,5-bis(alkylthio)pyridines from 2,6-diaminopyridine. *J. Chem. Soc. Chem. Commun.* **23**, 1139 (1980).

2 Ganesan, P. et al. Tetrahedral n-type materials: Efficient quenching of the excitation of p-type polymers in amorphous films. *J. Am. Chem. Soc.* **127**, 14530 (2005).

3 Gao, C. et al. Isostructural Three-Dimensional Covalent Organic Frameworks. *Angew. Chem. Int. Ed.* **58**, 9770–9975 (2019).

4 Gao, C., Li, J., Yin, S., Sun, J. L. & Wang, C. Twist Building Blocks from Planar to Tetrahedral for the Synthesis of Covalent Organic Frameworks. *J. Am. Chem. Soc.* **142**, 3718–3723 (2020).

5 Smeets, S., Wang, B., Cichocka, M. O., Angstrom, J. & Wan, W. Instamatic (Version 0.6) Zenodo (2018). https://doi.org/10.5281/zenodo.1217026

6 Kabsch, W. Integration, scaling, space-group assignment and post-refinement. *Acta Cryst.* **D66**, 133–144 (2010).

7 Wan, W.; Sun, J. L.; Su, J.; Hovmoller, S. & Zou, X. D. Three-dimensional rotation electron diffraction: software RED for automated data collection and data processing. *J. Appl. Crystallogr.* **46**, 1863–1873 (2013).

8 Sheldrick, G. M. SHELXT-Integrated space-group and crystal-structure determination. *Acta Cryst.* **A71**, 3–8 (2015).

9 Coelho, A. A. TOPAS and TOPAS-Academic: an optimization program integrating computer algebra and crystallographic objects written in C++. *J. Appl. Cryst.* **51**, 210–218 (2018).

10 Anwar, F.; Khaleel, M.; Wang, K. & Karanikolos, G. A. Selectivity Tuning of Adsorbents for Ethane/Ethylene Separation: A Review. *Ind. Eng. Chem. Res.* **61**, 12269 (2022).

11 Yang, L. et al. Adsorption in reversed order of C2 hydrocarbons on an ultramicroporous fluorinated metal-organic framework. *Angew. Chem. Int. Ed.* **61**, e202204046 (2022).

12 Gu, X. et al. Immobilization of Lewis Basic Sites into a Stable Ethane-Selective MOF Enabling One-Step Separation of Ethylene from a Ternary Mixture. *J. Am. Chem. Soc.* **144**, 2614 (2022).

13 Lin, R. et al. Boosting Ethane/Ethylene Separation within Isoreticular Ultramicroporous Metal–Organic Frameworks. *J. Am. Chem. Soc.* **140**, 12940 (2018).

14 Li, B. et al. Ethane/ethylene separation in a metal-organic framework with iron-peroxo sites. *Science* **362**, 443 (2018).

15 Chen, Y. et al. An ethane-trapping MOF PCN-250 for highly selective adsorption of ethane over ethylene. *Chem. Eng. Sci.* **175**, 110 (2018).

16 Liao, P.; Zhang, W.; Zhang, J. & Chen, X. Efficient purification of ethene by an ethane-trapping metal-organic framework. *Nat. Commun.* **6**, 8697 (2015).

17 Qazvini, O. T. et al. A Robust Ethane-Trapping Metal–Organic Framework with a High Capacity for Ethylene Purification. *J. Am. Chem. Soc.* **141**, 5014 (2019).

18 He, C. et al. Microregulation of Pore Channels in Covalent-Organic Frameworks Used for the Selective and Efficient Separation of Ethane. *ACS Appl. Mater. Interfaces* **12**, 52819 (2020).

19 Jin, F. et al. Bottom-Up Synthesis of 8-Connected Three-Dimensional Covalent Organic Frameworks for Highly Efficient Ethylene/Ethane Separation. *J. Am. Chem. Soc.* **144**, 5643 (2022).

20 Materials Studio, Version 7.0. (Accelrys Software Inc., San Diego, CA, 2016).

21 Van de Vondele, J.; Krack, M.; Mohamed, F.; Parrinello, M.; Chassaing, T.; Hutter, J. Fast and accurate density functional calculations using a mixed Gaussian and plane waves approach. *Comput. Phys. Commun.* **167**, 103 (2005).

22 Perdew, J.; Burke, K.; Ernzerhof, M. Generalized Gradient Approximation Made Simple. *Phys. Rev. Lett.* **77**, 3865 (1996).

23 Van de Vondele, J.; Hutter, J. Gaussian basis sets for accurate calculations on molecular systems in gas and condensed phases. *J. Chem. Phys.* **127**, 114105 (2007).

24 Goedecker, S.; Teter, M.; Hutter, J. Fluctuation formula for elastic constants. *Phys. Rev. B* **54**, 1 (1996).

25 Grimme, S.; Antony, J.; Ehrlich, S.; Krieg, H. A consistent and accurate ab initio parametrization of density functional dispersion correction (DFT-D) for the 94 elements H-Pu. *J. Chem. Phys.* **132**, 154104 (2010).
